# Digital Interventions for Depression and Anxiety in Older Adults: Protocol for a Systematic Review

**DOI:** 10.2196/22738

**Published:** 2020-12-23

**Authors:** Indira Riadi, Lucy Kervin, Kelly Teo, Ryan Churchill, Theodore D Cosco

**Affiliations:** 1 Gerontology Research Centre Simon Fraser University Vancouver, BC Canada; 2 Oxford Institute of Population Ageing University of Oxford Oxford United Kingdom

**Keywords:** systematic review, digital intervention, mental health, depression, anxiety, older adults

## Abstract

**Background:**

There is a high prevalence of older adults experiencing depression and anxiety. In response to heightened demands for mental health interventions that are accessible and affordable, there has been a recent rise in the number of digital mental health interventions (DMHIs) that have been developed and incorporated into mental health treatments. Digital interventions are promising in their ability to provide researchers, medical practitioners, and patients with personalized tools for assessing behavior, consultation, treatment, and care that can be used remotely. Reviews and meta-analyses have shown the benefits of DMHIs for the treatment and prevention of depression, anxiety, and other mental illnesses, but there is still a lack of studies that focus on the benefits and use of DMHIs in the older population.

**Objective:**

The aim of this systematic review is to investigate the current evidence for the effect of technology-delivered interventions, such as smartphone/tablet applications, remote monitoring and tracking devices, and wearable technology, for the treatment and prevention of depression and anxiety in adults older than 50 years.

**Methods:**

The academic databases SCOPUS, PsycINFO, AgeLine (EBSCO), and Medline (PubMed) will be searched from January 1, 2010, to the date of search commencement to provide a review of existing randomized controlled trial studies. The search will include 3 key concepts: “older adults,” “digital intervention,” and “depression/anxiety.” A set of inclusion criteria will be followed during screening by two reviewers. Data will be extracted to address aims and objectives of the review. The risk of bias for each study will be determined using appropriate tools. If possible, a random-effects meta-analysis will be performed, and the heterogeneity of effect sizes will be calculated.

**Results:**

Preliminary searches were conducted in September 2020. The review is anticipated to be completed by April 2021.

**Conclusions:**

The data accumulated in this systematic review will demonstrate the potential benefits of technology-delivered interventions for the treatment of depression and anxiety disorders in older adults. This review will also identify any gaps in current studies of aging and mental health interventions, thereby navigating a way to move forward and paving the path to more accessible and user-friendly digital health interventions for the diverse population of older adults.

**Trial Registration:**

PROSPERO International Prospective Register of Systematic Reviews CRD42020192532; https://www.crd.york.ac.uk/prospero/display_record.php?ID=CRD42020192532

**International Registered Report Identifier (IRRID):**

PRR1-10.2196/22738

## Introduction

Among older adults, 1 in 5 suffer from one or more mental disorders, with the numbers climbing as the world’s population gets older [1]. Recent literature has shown that older adults are highly vulnerable to mental disorders such as depression and anxiety [2,3]. The scarcity of access to mental health treatments and care for older adults in North America further diminishes help-seeking behaviors and increases the mental health stigma of the population [4], thereby deteriorating the already-low mental health literacy of many individuals [5]. Many seniors do not recognize a need for treatment but rather believe that these depressive and anxious feelings are normal, a side effect of growing old [6]. Additionally, older adults who do recognize a need for mental health services often face multiple barriers to accessing care, such as having limited knowledge regarding available services, few monetary resources, lack of access to transportation, and an overall negative view of mental illness [7,8].

Over the last several years, there has been an increase in popularity and availability of mobile digital technologies, which has triggered and pushed the development of mobile digital mental health interventions (DMHIs) including smartphone applications, remote monitoring and tracking devices, and wearable computers such as smartwatches and virtual/augmented reality headsets [9]. The World Health Organization, the United Kingdom’s National Health Service, and the US National Institute of Mental Health have recently identified smartphone, desktop, and tablet apps as efficient, cost-effective, and valuable methods to provide accessible treatments for mental disorders such as depression and anxiety [10].

Emerging research has found that DMHIs can be utilized for early identification, diagnosis, management, and analysis of adult mental health patients [11,12]. However, there are significantly fewer studies that focus on older adults, who have unique needs and preferences when it comes to technology-based health interventions [13]. The management of daily activities within the lives of older adults are extremely complex, as the majority of the older population must also deal with a multitude of late-onset chronic diseases [14]. Moreover, vulnerable older adult populations, such as those who live in rural areas, are suffering from a shortage of mental health care delivery due to the lack of care facilities, mental health professionals, and services in nonurban areas [15]. It is therefore important to acknowledge that the use of DMHIs is not confined within primary mental health care and facilities, but may also be used by individuals, in communities, and in senior centers. For example, technology can provide seniors, individually or in a group, access to participate in physical activities using motion sensors or other exergaming technologies, which can alleviate symptoms of depression and anxiety [16] and improve their general perceived wellness and quality of life [17]. Furthermore, improvement of mental health and quality of life can be a secondary outcome of using tech-based services that are based on individual needs, including various service deliveries [18], access to transportation [19], and the ability to participate in teleconferencing, distance education, and socialization [20].

DMHI research has the potential to spearhead a breakthrough in disease/illness intervention research as it has the ability to reach populations who otherwise might not engage in standard mental health interventions, expand the boundaries of the types of services available, and overcome geographic barriers through delivery of services to remote areas [21]. Despite the promises that DMHIs offer, there is the danger that comes from the digital divide. Older adults may feel unfamiliar with and excluded by new technologies, which may in turn exacerbate their feelings of inequitable healthcare, aggravate help avoidance behaviors, or even trigger self-deprecating feelings [22,23]. 

This systematic review aims to investigate the evidence for the effect of DMHI for the treatment and prevention of depression and anxiety in adults older than 50 years. We hope to not only understand the effectiveness of the most current DMHIs on older adults, but also gain insight into the various designs of interventions to understand what digital methods are accessible and more accepted by the older population.

## Methods

### Study Design

This systematic review protocol has been prepared following the PRISMA-P (Preferred Reporting Items for Systematic Reviews and Meta-Analyses for Protocols) 2015 checklist [24] (Multimedia Appendix 1). This review will gather recent studies to explore the effect of DMHIs in the prevention and treatment of depression and anxiety in adults older than 50 years.

### Search Methods

The academic databases SCOPUS, PsycINFO, AgeLine (EBSCO), and Medline (PubMed) will be searched for publications between January 2010 and the date of commencement. DMHIs for the treatment of depression and anxiety in older adults do exist prior to 2010 [25]. The reason behind this cutoff is that this review is focused on a modern and fast-paced topic. With the current production rate of new technologies, older studies using decade-old technologies are unlikely to still be relevant. A systematic search of each database will be conducted using a combination of search terms relating to the mental health problems targeted (depression and anxiety disorders), the means of intervention delivery (via the internet, using smartphones, using tablets, etc), population age (older adults), and the study design (RCT). Table 1 contains the full list of search strings to be input into each database search. Reference lists of included articles will also be screened for potentially relevant studies.

**Table 1:** Complete list of terms to be input into each database, separated into clusters of main key terms.

Cluster	Search items
A (older adults)	“older adult$” OR “senior$” OR “elderly” OR “elder$” OR “aged 50” OR “50+”
B (intervention)	“intervention$” OR “treatment$” OR “therapy”
C (mental health)	“mental health” OR “mental illness” OR “mental disorder$” OR “psychiatric illness*” OR “depress*” OR “anxiety”
D (technology)	“technol*” OR “digital” OR “online” OR “internet” OR “mobile” OR “electronic*” OR “social media” OR “smartphone$” OR “web*” OR “mobile app*” OR “smartphone app*” OR “VR” OR “AR” OR “virtual reality” OR “augmented reality” OR “wearable tech*” OR “wearable$” OR “computer*” OR “personal digital assistant$” OR “PDA$” OR “laptop$” OR “e-reader$” OR “Enterprise digital assistant$” OR “EDA$”

### Screening and Selection Process

All search results from the aforementioned databases will be downloaded into a reference management software in order to identify duplicates and allow for an easier screening procedure. First, all article titles and abstracts will be screened by two reviewers independently based on the inclusion and exclusion criteria. The results will then be discussed, and any disagreement will be resolved through consensus-based discussion or by a third reviewer. Next, full texts will be examined. Any disagreement between two reviewers will be resolved by discussion with a third reviewer.

### Eligibility Criteria

This review will include RCTs of digitally delivered mental health interventions for use by adults older than 50 years for their depressive or anxiety disorders. We will also include DMHIs that were used by caregivers, family members, and close relationships to aid or provide support for older adults with depressive or anxiety disorders. For the purposes of this review, DMHIs will be defined as interventions that are delivered using devices that have wireless cellular communication capability or are able to run software applications. We will therefore include interventions using smartphones, enterprise digital assistants, personal digital assistants, portable media players, video game consoles (including virtual/augmented reality headsets), desktop computers, laptops, tablets, and e-readers. Studies will be included if the sample population was assessed pre-intervention and post-intervention for anxiety or depressive symptoms by a clinician or assessed by the research team through the use of an anxiety or depression questionnaire. Studies that used self-administered depression/anxiety questionnaires that were completed online or by paper without the presence of a physician, psychologist, or a member of the research team will also be included. Only RCT studies will be included. There will be no restrictions placed on the theoretical basis of the interventions. We will include peer-reviewed papers in all languages and from all countries. Non–peer-reviewed articles, conference proceedings, case reports, editorials, opinion papers, and letters, as well as studies that focus on children or young adults, will be excluded.

### Data Extraction Process

We will extract the following data from each eligible study, prior to full-text extraction, into a bespoke Microsoft Excel (Microsoft Corporation) template: author or authors, aims, study design, participant information, sampling/recruitment methods, number of participants, the intervention (including the length of the interventions; the number of interventions, if multiple interventions were used; and the form or forms of delivery), and the author’s results or conclusion for the interventions. Participant information will include sample age and mental health status prior to the intervention (assessed using a depression/anxiety questionnaire).

### Risk of Bias Assessment

The risk of bias (ROB) will be assessed using the revised Cochrane risk-of-bias tool for randomized trials, version 2 (RoB 2) [26]. ROB will be evaluated for each bias domain listed in the RoB 2 assessment tool, which covers (1) bias arising from the randomization process, (2) bias due to deviations from intended interventions, (3) bias due to missing outcome data, (4) bias in measurement of the outcome, and (5) bias in selection of the reported result. The ROB will be conducted by the primary author and then checked by two reviewers independently. Disagreements will be discussed and resolved by a third reviewer. Studies will be assigned either “low risk,” “unclear risk,” or “high risk” status for each of the aforementioned 5 domains of bias.

### Data Analysis

A narrative synthesis of the findings from the included studies will be provided. This will describe the studies according to the following characteristics: (1) the components and method of delivery of interventions (eg, what type of device was used, was it delivered remotely or in person, what was the length of the study); (2) the characteristics of the sample population (mean age, gender, ethnicity, and socioeconomic status) and, if available, their views on mental health and mental health interventions; (3) the aim of the intervention (prevention or treatment of depression/anxiety, or both); (4) the types of outcome (eg, change in overall quality of life, change in depression/anxiety questionnaire answers, or structural changes in the brain observed using neuroimaging techniques); and (5) the longevity of outcome or the outcome of a follow-up study (were the subjects able to use these interventions beyond the observation period?).

If sufficient data is available, a random-effects meta-analysis will be conducted using the standardized mean differences to calculate effect size (Hedges *g*). Heterogeneity of effect sizes will then be calculated using the Q statistic and *I*^2^ statistic. Significant heterogeneity can be deciphered from a significant Q statistic, indicating more variation in effect sizes (which can solely be attributed to chance). *I*^2^ statistics will show heterogeneity as a percentage, with values of 25%, 50%, and 75%, which are associated with low, moderate, and high heterogeneity, respectively [27,28]. If possible, subgroup analyses will be conducted to examine the influence of the control conditions and the severity of mental health problems. We will assess for the presence of publication bias by displaying and examining the symmetry/asymmetry of a funnel plot of all studies included [29]. Furthermore, we will be using the GRADE (Grading of Recommendations Assessment, Development and Evaluation) framework to evaluate the quality of evidence for individual outcomes [30]. Sensitivity analysis will be performed following the Cochrane handbook recommendation by taking the poorest-quality evidence to determine its effects on the meta-analysis.

## Results

As of October 21, 2020, we have conducted preliminary searches on the aforementioned academic databases. Title and abstract screening will commence in December 2020, and full text screening will follow shortly after. We expect to begin data analysis in February 2021. The review is anticipated to be completed by April 2021.

## Discussion

DMHIs have the potential to offer unique and innovative opportunities for the treatment of mental health in older adults. Developers and medical practitioners in the mental health field can use the advantages of digital innovations to provide older adults with helpful instruments that are not limited by the patient/user’s geographical location. This systematic review will offer insight into the potential benefit of technology-delivered programs and provide up-to-date information on existing DMHIs for older adults through the exploration of current digital interventions for the treatment of depression and anxiety. This systematic review also hopes to identify any gaps in current studies of mental health interventions and the aging population, thereby navigating a path for DMHI research to move forward.

The fundamental advantage of DMHI is the opportunity it brings to blur the lines of inequality when it comes to the accessibility of mental health resources. However, currently, there are very few studies looking at the effects of DMHIs in populations that specifically face significant social and physical challenges. Within the (already vulnerable) older adult population, there exists a subpopulation of more vulnerable older adults such as, but not limited to, those living in abusive families, the homeless, the poor, racial minorities, refugees, gender/sexual minorities, and people with physical disabilities and chronic diseases. Schueller et al [31] have written extensively about how digital interventions can provide opportunities to alleviate mental health disparities among marginalized populations, stating that technology can be tailored to be culturally sensitive and low cost, and can also overcome barriers of time, place, and language. Despite this progress, it has been understood that each subpopulation of marginalized older adults has differing strengths and needs when it comes to using DMHIs. Extensive work still needs to be done in order to translate the potential of technology to address mental health needs among diverse, marginalized older populations into reality.

## Appendix

Multimedia Appendix 1 PRISMA-P Checklist.

## Abbreviations

DMHI: digital mental health intervention
GRADE: Grading of Recommendations Assessment, Development and Evaluation
PRISMA-P: Preferred Reporting Items for Systematic Reviews and Meta-Analyses for Protocols
RCT: randomized controlled trial
ROB: risk of bias
RoB 2: risk-of-bias tool for randomized trials, version 2
